# Fibrillary glomerulonephritis disease natural history and outcomes: a retrospective two centre cohort study

**DOI:** 10.1186/s12882-025-04187-z

**Published:** 2025-07-01

**Authors:** Yimeng Zhang, Jyoti Baharani, Bamidele Ajayi, Jennifer Pinney

**Affiliations:** University Hospitals Birmingham NHS Foundation Trust Queen Elizabeth Hospital Birmingham, Mindelsohn Way, Birmingham, B15 2GW UK

**Keywords:** Fibrillary glomerulonephritis, Rare diseases, Disease progression, Immunosuppression treatment, Renal histology

## Abstract

**Background:**

Fibrillary glomerulonephritis (FGN) is a rare immune complex-mediated glomerulonephritis characterised by the deposition of anomalous fibrillary structures within the glomeruli.

The prognosis for patients with FGN is usually poor with rapid progression to end stage kidney disease (ESKD). There are currently limited data to suggest an optimal therapy strategy to prevent this. Most case series describing FGN come from North America with limited research from the UK.

**Methods:**

This is a retrospective case series of patients who presented with biopsy proven FGN to two renal centres within the West Midlands, between 2006 and 2022.

**Results:**

Twenty-one patients with a histological diagnosis of FGN were identified within the 16-year period. Median eGFR at the time of biopsy was 29 mL/min/1.7 (IQR 18–55), serum albumin 31 g/L (IQR 28–33) and ACR was 368 mg/mmol (IQR 303–596). The median follow-up for the cohort was 50 months (range 12–138). DNAJB9 staining was done for five patients, all were positive.

Immunosuppression was used in 8 patients following diagnosis of FGN. Treatment varied between steroid, rituximab and cyclophosphamide. Patients with crescents on the biopsy were more likely to receive a trial of immunosuppression. Progression to ESKD was common, 7 (33%) patients required renal replacement therapy within 12 months of diagnosis of FGN.

**Conclusions:**

To date, there are limited numbers of case series of FGN due to the rare nature of the disease. We describe the natural history of this rare kidney condition, and highlight the challenges faced by clinicians where evidence for successful therapeutic options is lacking.

## Key learning points

**What was known:**

- Fibrillary glomerulonephritis (FGN) is a rare immune complex-mediated glomerulonephritis first described in 1977 by Rosenmann and Eliakim, in those with unique histological findings of fibrillary structures on renal biopsy.

- Staining for DNA heat shock protein family (Hsp40) member B9 (DNAJB9) has 98–100% sensitivity and > 99–100% specificity as a biomarker for the diagnosis of FGN. It is not found in healthy glomeruli or other comparable glomerular diseases.

- There are currently limited data to suggest an optimal therapeutic strategy to prevent renal function decline with trials of steroids along with immunomodulation treatments demonstrating inconclusive results.

**This study adds:**

- We describe the natural history of fibrillary glomerulonephritis and highlight the challenges faced by clinicians where evidence for successful therapeutic options is lacking.

- In our case series, 38% of patients received immunosuppressive therapy with some demonstrating a slower decline in eGFR over time following treatment.

- This case series further highlights the poor overall prognosis of this condition with a significant number (33%) of patient requiring renal replacement therapy within 12 months of diagnosis and an average eGFR decline of 1 ml/min.

**Potential impact:**

- All of the histology samples from our case series which were tested for DNAJB9 yielded a positive result. Future research on serum and urine levels of DNAJB9 may provide a non-invasive way of diagnosing FGN.

- There is very limited evidence that immunosuppressive therapy is effective in slowing the progression of FGN, our study highlights the potential benefit of this treatment in selected patients, and this warrants further evaluation.

- The study highlights the grim prognosis of FGN with rapid progression to ESKD, we urge for larger scale studies with global or national registry data to obtain more powerful results.

**Clinical trial number:**

Not applicable.

## Introduction

Fibrillary glomerulonephritis (FGN) is a rare immune complex-mediated glomerulonephritis. FGN was first described in 1977 by Rosenmann and Eliakim who published a case report of a patient with nephrotic syndrome and unique histological findings of fibrillary structures on a renal biopsy [1]. FGN is characterised by the deposition of anomalous fibrillary structures within the glomeruli. These fibrils, composed predominantly of IgG or polyclonal immunoglobulins and complement components, incite chronic inflammation and progressive damage to the renal parenchyma. FGN is known to typically occur in the 5th decade with the characteristic presentation of nephrotic syndrome, hypertension, reduced renal function and microscopic haematuria [2, 3]. FGN may be idiopathic or associated with a monoclonal gammopathy, hepatitis C infection, malignancy or autoimmune diseases [3–5].

The definitive diagnosis is made on a renal biopsy, enabling histopathological identification of the distinctive fibrillary deposits within the glomerular mesangium and peripheral capillary loops. Typical electron microscopic findings are of organised, randomly orientated and non-branching fibrils of an average of 20 nm in thickness, in the mesangium and/or along the glomerular basement membranes (GBM). FGN deposits typically stain negative for Congo red, which distinguishes the condition from amyloidosis. The glomerular deposits are considered to be composed of a complex of antibodies and antigens as they usually positively stain for kappa and lambda (85–95%), as well as IgG (up to 100%), IgM (47–56%), IgA (28–39%), C1q (65%) and C3 (91–92%) [2, 3, 6]. FGN can demonstrate a membranoproliferative pattern on light microscopy, this was reported in 44% of cases, with mesangial proliferation and expansion, along with GBM duplication and endocapillary proliferation [7]. Dasari et al. used mass spectrometry to identify DNA heat shock protein family (Hsp40) member B9 (DNAJB9) as the fourth most abundant protein in FGN glomeruli [8]. Staining for DNAJB9 has 98–100% sensitivity and > 99–100% specificity as a biomarker for the diagnosis of FGN. It is not found in healthy glomeruli or other comparable glomerular diseases [9].

The prognosis for patients with FGN is usually poor with rapid progression to end stage kidney disease (ESKD) [5]. There are currently limited data to suggest an optimal therapeutic strategy to prevent this. Management strategies primarily focus on mitigating symptoms and supportive treatment, with a focus on blood pressure management and reduction of proteinuria using conventional methods such as angiotensin converting enzyme (ACE) inhibition and more recently the use of sodium-glucose co-transporter-2 (SGLT2) inhibitors.

FGN can be associated with a potential driver such as a monoclonal protein, when this is the case it is termed a monoclonal gammopathy of renal significance (MGRS) and this requires treatment of the underlying clone. Similarly, where there is an alternative driver such as infection or an immune mediated condition treatment of the underlying condition is recommended. In idiopathic FGN immunosuppressive treatment has been trialled, most commonly with steroids, with or without a second agent such as cyclophosphamide or rituximab, with inconclusive results [3, 10, 11]. Following renal transplantation, FGN has been shown to reoccur in 21–36% of patients and may result in allograft failure [3, 12].

FGN is identified in less than 1% of all renal biopsies, which explains the lack of published evidence on the management and prognosis of the condition [13]. The majority of case series describing FGN come from North America [3, 4, 7, 14, 15] with limited research from the UK. Here we report a case series of FGN cases from two multicultural renal centres within the West Midlands.

## Methods

Patients were identified as having a coded diagnosis of FGN between 2006 and 2022 via histopathological databases at two renal centres within the West Midlands. The diagnosis was made following review of native renal biopsy samples using light microscopy, immunofluorescence and electron microscopy by onsite renal histopathologists. Tissue samples were examined by specialist renal pathologists who followed the standard published protocol [16]. Immunofluorescence was performed on Roche Benchmark Ultra platforms, using direct immunofluorescence method. The antibodies used for the renal staining panel were: C3, C1q, IgA, IgG, IgM, Kappa and Lambda.

Retrospective data collection of demographic data, baseline characteristics, blood results, comorbidities, immunosuppression treatment and time to ESKD were recorded from the electronic patient records. Results available within one month of renal biopsy were included in the baseline characteristics. In individuals where only urine protein creatinine ratio (PCR) results were available, values were converted to urine to albumin creatinine ratio (ACR) level by multiplying by 0.7 as per guidance from the UK Kidney Association (https://www.ukkidney.org/health-professionals/information-resources/uk-eckd-guide/measurement-kidney-function).

Data were analysed using Microsoft Excel 2010, Statistical significance was assumed at *P* < 0.05.

## Results

### Demographics and presentation

Twenty-one patients with a histological diagnosis of FGN were identified within the 16-year period (2006 to 2022). At the time of native renal biopsy, the median eGFR was 29mL/min/1.7 (IQR18-55), serum albumin 31 g/L (IQR28-33) and ACR 368 mg/mmol (IQR 303–596). The median follow-up for the cohort was 50 months (range 12–138). Table 1 describes the baseline characteristics of the cohort at the time of renal biopsy.

**Table 1:** Baseline characteristics of patients diagnosed with FGN at the time of renal biopsy and time to renal replacement therapy

			Number of patients (%)
Gender	Male		11 (52%)
	Female		10 (48%)
Ethnicity	British Caucasian		17 (81%)
	Caucasian – other		0
	Asian		0
	Afro-Caribbean		0
	Not documented		4 (19%)
Age (years)	Mean ± SD		63 ± 11
Associated medical conditions	Hypertension		15 (71%)
	Type 2 diabetes mellitus		5 (24%)
	Graves thyrotoxicosis		1 (5%)
	Idiopathic thrombocytopenic purpura		1 (5%)
	Cutaneous vasculitis		1 (5%)
	Malignancy		
	Prostate cancer		1 (5%)
	Chronic lymphocytic leukaemia		1 (5%)
	Monoclonal gammopathy of undetermined/renal significance		3 (14%)
	Hepatitis B		0
	Hepatitis C		0
Presenting complaint to renal team	Decline in renal function		7 (33%)
	Oedema		6 (28%)
	Proteinuria		4 (19%)
	Haematoproteinuria		3 (14%)
	Shortness of breath		1 (5%)
Laboratory results at time of biopsy	Medium eGFR (*n* = 21)		29mL/min/1.73m2 (IQR 25–78)
	Median serum albumin (*n* = 21)		31 g/L (IQR 28–33)
	Median urine ACR mg/mmol (*n* = 17)		368 (IQR 303–596)
	Microscopic haematuria (*n* = 15)		10 (67%)
	Abnormal Kappa: lambda ratio < 0.26 or > 1.65 (*n* = 17)		4 (24%)
	Urine Bence-Jones Protein present (*n* = 3)		1 (33%)
	Monoclonal immunoglobulins (*n* = 19)	IgG	2 (11%)
		IgM	1 (5%)
Time to renal replacement therapy (months) (*n* = 10)	≤ 6 months		5 (50%)
	6–12 months		0
	12–24 month		2 (20%)
	> 24 months N		3 (30%)

### Histological findings

Table 2 displays the histological findings on renal biopsy. DNAJB9 was not available for earlier samples, 5 (24%) of the total 21 histological samples had DNAJB9 immunohistochemistry staining, all were positive. The two patients with lambda predominance on immunohistochemistry did not have a detectable lambda clone in serum or urine. One patient had a small clonal IgM lambda paraprotein present, their immunofluorescence demonstrated equal positivity for kappa and lambda.

**Table 2:** Histological findings on renal biopsy in patients with fibrillary glomerulonephritis

			Number of patients (%)
Light microscopy (*n* = 21)	Mesangial expansion		14 (67%)
	Endocapillary proliferation		6 (29%)
	GBM thickening		6 (29%)
	Crescents		6 (29%)
Fibril size (*n* = 19)			Range 8–30 nm
DNAJB9 stain (*n* = 5)	Positive		5 (100%)
	Negative		0
Congo red stain (*n* = 20)	Positive		0
	Negative		20 (100%)
Immunofluorescence (*n* = 19)	Kappa & Lambda	L = K	3 (16%)
		L > K	2 (11%)
		K > L	0
	IgG		18 (90%)
	IgM		5 (26%)
	IgA		7 (36%)
	C1q		5 (26%)
	C3		18 (95%)

### Treatment

Immunosuppression was used in 8 patients following diagnosis of FGN. Patients who received a trial of immunosuppression had worse renal function at the time of biopsy (median eGFR 26 ml/min in the immunosuppressed group vs. 47 ml/min in the non-immunosuppressed group). Median albumin level was no different (31 g/L) in both groups. More patients with crescents on their biopsy received immunosuppression compared to those without crescents (83% versus 13%). 5 out of 8 (63%) patients who received immunosuppression had crescents seen on biopsy compared to 1 out of 13 (8%) patients who did not have a trial of immunosuppression.

Treatment was varied, four patients received high dose steroids only; two of which intended to treat FGN (3 doses of 500 mg IV methylprednisolone in one, the other received a tapering course of prednisolone from 60 mg once daily over a three month period). One patient received intravenous methylprednisolone followed by a course of prednisolone 30 mg once daily to treat an underlying immune thrombocytopenia. The last patient was treated with prednisolone 15 mg once daily for polymyalgia rheumatica. Rituximab was used in 5 patients, 2 of which were initiated for a primary haematological condition (chronic lymphocytic leukaemia and MGRS). One patient was given cyclophosphamide plus prednisolone.

### Outcome

**Fig. 1:**
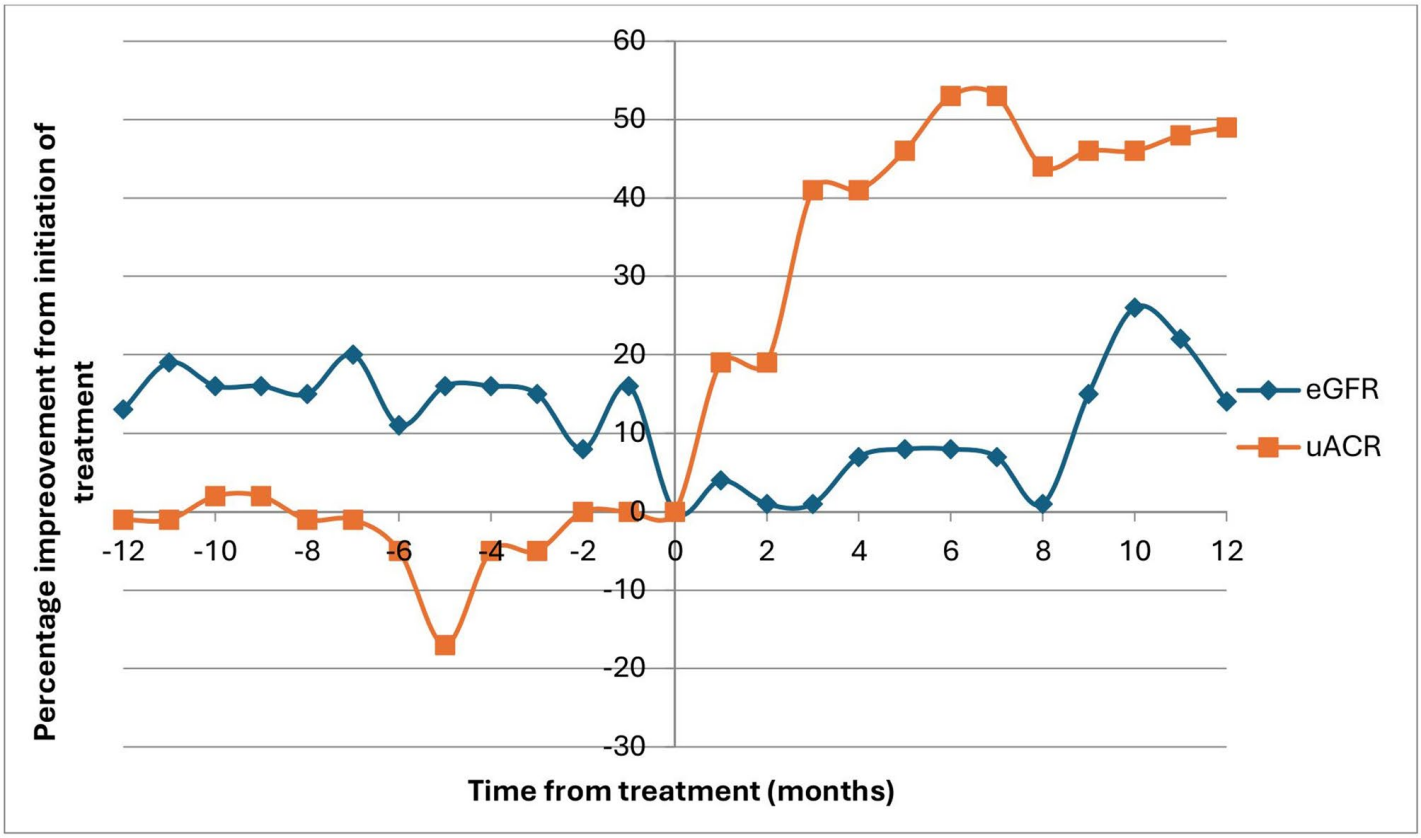
Urine ACR vales before and following initiation of treatment (time 0) over a 24-month period for the 8 patients who have received immunosuppression treatment, one patient not graphed as did not have proteinuria documented at time of biopsy and no urine ACR or PCR results available

Progression to ESKD was common, with 7 (33%) patients requiring renal replacement therapy within 12 months of diagnosis of FGN. Out of the 8 patients who received high dose steroids and/or rituximab or cyclophosphamide, 2 (25%) required dialysis within 12 months of diagnosis. 3 out of the 8 patients (38%) demonstrated improvement in renal function (eGFR more than doubled in all cases) following immunosuppression treatment, all of which had crescents demonstrated on biopsy. Two did not demonstrate any improvement and 3 did not have significant data available to evaluate response. The percentage change in renal function and proteinuria 12 months prior and following immunosuppression treatment are displayed in Fig. 1. Out of the two patients had a decline in urine ACR following treatment, one was given rituximab and bendamustine for underlying chronic lymphocytic leukaemia, the other was given two doses of rituximab without having an underlying clone.

**Fig. 2:**
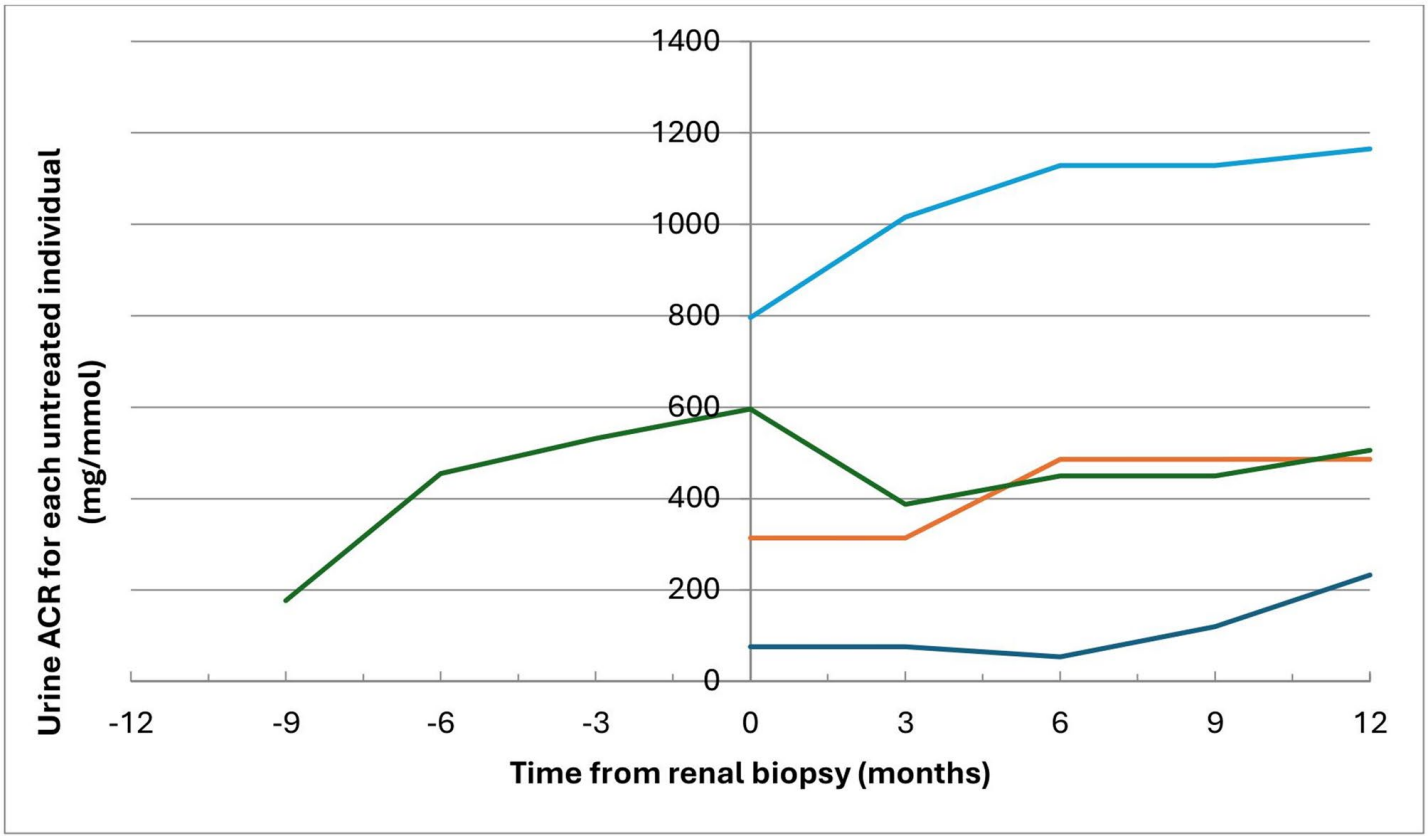
Urine ACR values before and following initiation of diagnosis/renal biopsy (time 0) over a 24-month period for the patients who have not received immunosuppression treatment

Figure 2 demonstrates the trend of urine ACR before and after diagnosis of FGN in those who did receive immunosuppression treatment. Five patients did not have sufficient number of urine ACR or PCR results, with two or less values over the 24-month period. Four patients were excluded as they were initiated on renal replacement therapy within 2 months of renal biopsy. All four patients included in this figure were diagnosed with idiopathic FGN.

**Fig. 3:**
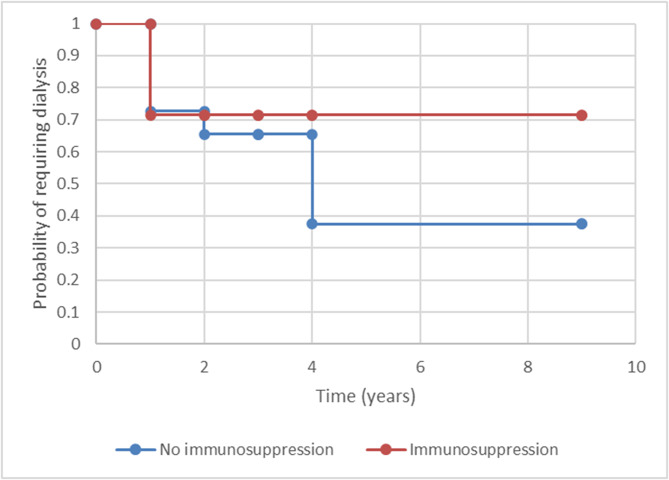
Kaplan Meier curve comparing time to initiation of dialysis between patients with or without immunosuppression treatment

There was no significant difference in time to initiation of dialysis in patients treated with or without immunosuppression, Fig. 3 (*n* = 21) (*p* = 0.32).

Of the patients who were not dialysed within one month of renal biopsy, the median rate of change in renal function was − 1 ml/min/year with a range of -34 to + 17 ml/min/year. There was no statistical difference in the mean rate of decline of eGFR per year in the treated and untreated groups, with a mean increase in eGFR of 8 ml/min/year for treated patients vs. decline of 3 ml/min/year in the untreated group (*p* = 0.16).

No patients died within 12 months of diagnosis. Five patients died during follow-up, the median time to death was 96 months (range 17–135).

## Discussion

We describe the natural history of this rare kidney condition, and highlight the challenges faced by clinicians where evidence for successful therapeutic options is lacking.

DNAJB9 was discovered in 2002 under a different name of endoplasmic reticulum-localised DNAJ homologs 4 (ERdj4) and was known for possible involvement in protein folding or endoplasmic reticulum associated degradation [17]. DNAJB9 has since been linked to FGN in 2017, where it was noted to be the fourth most abundant protein in the FGN glomeruli. DNAJB9 is not found in normal glomeruli hence it is an incredibly specific biomarker for FGN [9]. The pathogenesis of DNAJB9 is not yet completely understood, with multiple hypotheses with regard to its upregulation [18]. This further highlights the gaps in our understanding of FGN. Immunochemistry testing for DNAJB9 has now replaced electron microscopy as the new gold standard for the diagnosis of FGN. All of the histology samples from our case series which were tested for DNAJB9 yielded a positive result. Future research on serum and urine levels of DNAJB9 may provide a non-invasive way of diagnosing FGN [18].

To date, there are limited numbers of case series due to the rare nature of the disease. Various publications have identified either a female or male predominance of FGN, with the majority of the patients reported of Caucasian ethnicity [15, 19]. In keeping with our findings, FGN is reported to mainly affect people between the ages of 49–60 years [19]. 14% of our patients had an autoimmune condition; in keeping with similar studies (10–30%) [3, 6, 11, 20]. Despite the known association between FGN and hepatitis C, none of our patients have been diagnosed with hepatitis C infection [15, 21, 22]. Underlying malignancy was typically identified in 16–23% of patients [3, 6], which is more frequent than those identified in our cohort. Recently published UK guidelines recommend an assessment for an underlying clone, with plasma and urine assessments in all patients with FGN. In those with no detectable clone and DNAJB9 positivity on their biopsy, it is not recommended for further haematological investigation or treatment [23].

There is very limited evidence that immunosuppressive therapy is effective in slowing the progression of FGN, with most reporting restricted or no improvement in kidney function, proteinuria or time to ESRD [3, 11, 24]. There is some research to suggest that rituximab is effective at decreasing proteinuria and preserving kidney function in FGN [10, 15, 25]. A recent prospective clinical trial demonstrated potential effectiveness of rituximab in preservation of renal function over 12 months, with no significant decline in proteinuria or DNAJB9 levels [26]. Sauvage, et al., demonstrated the possible benefit of a combination of rituximab, oral cyclophosphamide and prednisolone in the preservation of kidney function when compared to the outcome data of other published cohorts [14]. The current data available on FGN remains inconclusive, with no large scale or randomised controlled trials. In our case series, 38% of patients received immunosuppressive therapy following a histological diagnosis of FGN. Those who were given immunosuppression had a lower baseline eGFR and more often had crescents on their renal biopsy. Some patients who received immunosuppression demonstrated a slower decline in eGFR over time following treatment. However, these findings did not reach significance, which is likely secondary to the small sample size limiting the statistical power. Patients in our case series were given varied treatments including steroid, rituximab and cyclophosphamide; therefore it is difficult to comment on the efficacy of one particular medication.

FGN is known to have a poor overall outcome and prognosis, with nearly half of the patients progressing to ESRD within 4 years of diagnosis [3, 6, 24]. Although, our figures cannot be compared directly, a significant number (33%) of patients in this case series required renal replacement therapy within 12 months of diagnosis, with an average eGFR decline of 1 ml/min.

The grim prognosis of FGN with rapid progression to ESKD and the substantial risk of disease recurrence post-renal transplantation necessitate the need for larger scale studies [12]. Given the rarity of this disease, a collaborative approach is crucial, with global or national registry required to obtain more powerful results. This will aid in the formulation of more evidence on the most effective treatment and enable greater understanding of the disease as a whole.

## Conclusion

The study’s limitations, such as its retrospective nature, small sample size, and the absence of standardised treatment regimens, accentuate the necessity for robust prospective studies. Future research should focus on elucidating the underlying mechanisms driving FGN pathogenesis, exploring targeted therapies to halt disease progression, and identifying prognostic markers to stratify patient outcomes. Prospective multicentre studies involving larger patient cohorts would provide deeper insights into FGN’s natural history, therapeutic responses, and long-term outcomes.

The observed decline in renal function despite attempted treatments, along with the mortality rate, underscores the urgency for collaborative efforts in clinical trials and translational research. Developing validated outcome measures, establishing therapeutic benchmarks, and exploring novel immunomodulatory strategies tailored to FGN’s unique pathophysiology should be prioritised. Additionally, integrating molecular profiling and advanced imaging techniques could offer a more nuanced understanding of disease progression and response to interventions. The research surrounding DNAJB9 revolutionised the diagnosis for FGN and provides hope in future targeted treatment for this rare condition.

## Data Availability

All data generated or analysed during this study are included in this published article.
